# Cerebral Malaria: Current Clinical and Immunological Aspects

**DOI:** 10.3389/fimmu.2022.863568

**Published:** 2022-04-20

**Authors:** Karin Albrecht-Schgoer, Peter Lackner, Erich Schmutzhard, Gottfried Baier

**Affiliations:** ^1^ Division of Translational Cell Genetics, Medical University of Innsbruck, Innsbruck, Austria; ^2^ Department of Neurology, Klinik Floridsdorf, Wien, Austria; ^3^ Department of Neurology, Medical University of Innsbruck, Innsbruck, Austria

**Keywords:** cerebral malaria (CM), CD8+, T cell sequestration, blood brain barrier (BBB), pathophysiology of CM, activation of the brain endothelium, parasite virulence factors, host virulence factors

## Abstract

This review focuses on current clinical and immunological aspects of cerebral malaria induced by *Plasmodium falciparum* infection. Albeit many issues concerning the inflammatory responses remain unresolved and need further investigations, current knowledge of the underlying molecular mechanisms is highlighted. Furthermore, and in the light of significant limitations in preventative diagnosis and treatment of cerebral malaria, this review mainly discusses our understanding of immune mechanisms in the light of the most recent research findings. Remarkably, the newly proposed CD8+ T cell-driven pathophysiological aspects within the central nervous system are summarized, giving first rational insights into encouraging studies with immune-modulating adjunctive therapies that protect from symptomatic cerebral participation of *Plasmodium falciparum* infection.

## Introduction

Malaria is a mosquito-borne infectious disease that is self-limiting even without therapy. However, in 1–2% of cases, mostly among children under the age of five, malaria becomes severe and life-threatening (1). Why young children are especially prone to develop severe and cerebral malaria (CM) is not fully understood. The vector responsible for most complicated malaria cases is the parasite *Plasmodium falciparum* (*Pf*). However, *P. vivax* infections also can cause CM (2), and *P. knowlesi* occasionally provokes severe malaria signs and symptoms (3).

Although it has become a declared global target of the *World Health Organisation (WHO)* to eliminate malaria worldwide and malaria cases and death have been reduced within the last ten years, in 2021, this progress was impeded as the COVID-19 pandemic disrupted malaria services and diagnosis. The immense number of malaria cases in 2021 (241 million cases) and the distressing number of deaths (627 000) revealed how fragile medical services are. Especially in the African region, where 96% of all malaria deaths occurred, 80% were children under five, significantly more effort is needed to reverse the last year’s trend for 2022 (4).

The definition of severe malaria by the *WHO* is *Pf* parasitemia together with one or more of the following medical conditions: impaired consciousness, prostration, multiple convulsions, acidosis, hypoglycemia, anaemia, renal impairment, jaundice, pulmonary oedema, significant bleeding and shock (5). Apart from infants, pregnant women are also more likely to develop severe malaria, particularly in the second and third trimesters. Placental malaria can lead to fetal and maternal death when untreated, and premature labour and children with low birth weight are common complications even with intermittent preventive treatment in pregnancy (4). Other susceptible population groups are non-semi-immune persons, e.g. travellers or migrant workers, moving to holo-endemic areas.

Cerebral malaria (CM) is characterized by unarousable coma not attributable to other neuro-pathologies combined with *Pf* parasitemia. Of all severe malaria complications, CM is the most prevalent and deadly, with a mortality rate of 100% if untreated and a patient fatality of 15-25% if treated with current first-line anti-malaria therapy (6). Children with CM present acutely in a coma often precipitated by seizure and a one to three-day history of fever, vomiting, and anorexia. Within two days, most children will have recovered from or succumbed to the disease (7). 14-25% of children recovering from CM suffer from long-term sequelae like cognitive and hearing impairments (8, 9). As there is no specific therapy beyond symptomatic neurocritical management, all strategies aim to eliminate the parasite from the system while targeting symptoms arising from severe complications like respiratory distress or convulsions. Furthermore, it is impossible to predict which children are likely to develop cerebral complications because the exact molecular mechanisms in human pathology remain unsolved. In animal studies, which are a reasonable way to investigate molecular pathophysiology, it has been shown that CM is, at least in part, an immune-mediated disease. Most likely, cerebral complications are caused by a misguided host immune response provoked by the parasite infection. Therefore adjunctive therapies focusing on the regulation of immune cells are interesting new treatment strategies (10).

## Treatment of Severe and Cerebral Malaria

*Artemisinins*, which have the fastest parasite clearing time of all anti-malarial drugs, have become the drugs of the moment, and *artesunate* is the first-line therapy for treating severe and cerebral malaria in both children and adults (5).

The main difference between the treatment of uncomplicated and severe malaria is the route of administration of the artemisinin-based therapy. Artesunate should be administered intravenously for 24 hours in severe and cerebral malaria cases. If an intravenous application is not possible, the intramuscular route is the second-best option, and artemether is recommended in case of missing artesunate. After that, a three-day oral artemisinin-based combination therapy (ACT) should be followed (5).

Beyond intravenous artesunate, no “brain-specific” drug is available. Symptomatic therapy strategies are the only possibility to manage organ manifestations and intracranial complications of *Pf* malaria. Although not widely available, respiratory support and artificial ventilation are crucial (11). Increasing intracranial pressure decreases cerebral perfusion and leads to secondary transtentorial herniation, the primary cause of death in children with CM (12). Seizure management is another critical treatment, as up to 70% of children with severe and cerebral malaria have seizures, which, when treated with benzodiazepines, are less likely to cause secondary neurological damage, and fever should be controlled to reduce high fever convulsions and long-term neurologic damage (12).

Although it is a breakthrough, the first-ever malaria vaccination only provides partial protection. A four-dose regimen (i.m.) of the MosquirixTM (RTS,S/AS01) vaccine showed protection from severe and cerebral malaria in 32.2% of children aged 5-17 months (13). Therefore, seasonal chemoprevention should continue in addition to vaccination (14).

## Genetic Coevolution

There have been millenniums of coevolution between humanity and *Plasmodium* species. Considering this, it is no surprise that both parasite and host have undergone genetic alterations for successful coexistence. On the host side, protective variations include sickle-cell anaemia (15), thalassemia (16), *G6PD* deficiency (17) and other haemoglobinopathies with high incidence in malaria-endemic countries. These severe inherited haemoglobin disorders are nicely reviewed elsewhere (18).

Several novel genetic variants have been identified with new technologies for genome-wide association studies (GWAS). Many genetic polymorphisms related to malaria are relevant for red blood cell (RBC) biology, like variants of the *ATP2B4* gene, a plasma membrane calcium transporter contributing to resistance against severe malaria (19), or variants of the *FREM3* gene and a cluster of three glycophorin genes (*GYPE, GYPB* and *GYPA*) associated with 33% protection against severe malaria (20). However, newly identified single nucleotide polymorphisms (SNPs) indicate the importance of other cells for malaria disease progression, as variations in genes encoding proteins necessary for neurons, endothelial cells and immune cell-signalling have been identified with GWAS. For example, a SNP close to the *DDC* (L-DOPA decarboxylase) gene is associated with cerebral malaria susceptibility (21). *DDC* is an enzyme that catalyzes the decarboxylation of L-DOPA to dopamine, which is involved in neuronal signalling and regulating the activation of specific lymphocyte subtypes (22). SNPs near the *MARVELD3* gene have been suggested to protect against the severe progression of malaria (19, 23). Structural variants of the gene product of *MARVELD3* or alterations in its expression could influence the barrier function and endothelial adherence of parasitized erythrocytes. A more recent GWAS identified the *IL-23R* and *IL-12RBR2* genes associated with alterations in malaria severity (24). Both genes are essential for immune cell signalling, especially for T-cells. Variants of *IL-12B* are protective against cerebral malaria in children (25). Polymorphisms in Fc gamma receptors on the surface of immune cells such as B-cells, NK cells and macrophages have been linked to resistance and susceptibility to malaria in different population studies (26).

Accumulating evidence indicates that the G protein-coupled signal transduction pathways are involved in the regulation of malaria, specifically in the severe, life-threatening manifestations of the disease. In a case-controlled study of adults, SNPs of *ADORA2A* and *GRK5* genes were associated with virulence and infectivity of the malaria parasite (27). The *ADORA2A* gene was associated with severe *Pf* malaria in children in a meta-analysis that evaluated several G protein-coupled signalling pathways (28). Identified genes and proteins listed in **Table 1** might be as new drug targets and require further exploration in malaria studies to validate and functionally characterize causalities to enhance our understanding of cerebral malaria pathology.

**Table 1:** Genetic variations and their association to malaria.

Genetic polymorphism	Cell type	Biological consequence	Association with malaria	Ref.
HBB (HbAS)	RBCs	Sickle cell trait (heterozygous) Sickle cell disease (homozygous)	Protective against malaria by increased clearance of sickling infected RBCs Increased susceptibility to severe malaria	(15, 18)
HBA1, HBA2	RBCs	α-Thalassemia, hetero- and	Protective against severe malaria and severe malaria anemia	(16, 18)
Glucose-6-phosphate dehydrogenase	RBCs	Glucose-6-phosphate dehydrogenase	Protective against severe malaria in female heterozygotes and male homozygotes because of decreased parasite invasion	(17, 18)
ATP284	RBCs	Encodes PMCA4 (ubiquitous Ca2+pump)	Expected to be protective against parasite invasion into RBCs	(19)
FREM3/GYPA,B,E	RBCs	Encode erythrocyte-binding and parasite binding ligands	Protective against severe malaria through	(20)
DDC	Neuronal cellslymphocytes	Encoding L-dopa decarboxylase	Increased susceptibility to severe and cerebral malaria	(21, 22)
MARVELD3	ECs	Encodes tight-junction structures of vascular endothelial cells	Suggested to be protective by maintaining endothelial barrier function	(19, 23)
IL23RIJL-12RBR2	Lymphocytes	Improved cell-mediated immune response against intracellular parasites	Protective against severe malaria	(24, 25)
FC gamma receptor	Lymphocytes	Altered immune response	Protective or increased susceptibility, depending on the SNP location	(26)
ADORA2A	RBCs Lymphocytes ECs	Encodes Adenosine 2a receptor	Increased susceptibility for severe malaria	(27)
GRK5	Lymphocytes	Encodes G-coupled receptor kinase 5	Increased susceptibility for severe malaria	(28)

## Pathophysiology of Cerebral Malaria

Magnetic resonance imaging has achieved vital progress in elucidating cerebral malaria pathology in children suffering from cerebral manifestations. It has become evident that brain swelling and brain stem herniation lead to respiratory arrest and death (6). Further investigations could link the swelling of the brain to a dysfunctional blood-brain barrier, indicating increased vascular permeability (29). Causative for the penetration of vascular fluid into brain tissue might be an excessive inflammatory response to sequestration of parasitized erythrocytes in the cerebral vasculature (30). Overall, CM seems to be caused by a combination of host factors and parasite factors, leading to inflammation and coagulation in the brain vasculature (31). Endothelial cell dysfunction due to inflammation is the main driver for brain pathology concerning host factors. On the parasite side, PfEMP-1 (*Pf* erythrocyte membrane protein-1) molecules on the surface of iRBCs are responsible for most parasite-host cell interactions.

## Parasite Virulence Factors

In order to escape the clearance by the spleen, iRBCs adhere to vessel walls in several organs (32). The surface molecule PfEMP-1 mediates this sequestration by binding to the host vasculature *via* interaction with specific adhesion molecules. PfEMP1 is encoded by the *var* gene family with approximately 60 members (33). The extracellular (interacting) part of PfEMP1 consists of Duffy-binding-like (DBL) domains and cysteine-rich interdomain regions (CIDR), each of which can be subdivided into seven and three main sequence classes, respectively (34). Among the host endothelial adhesion molecules, intercellular adhesion molecule-1 (ICAM-1) has long been suggested as the main anchor point for PfEMP-1 (35). Still, more recently, CD36 and endothelial protein C receptor (EPCR) were identified as more probable binding sites (36, 37). CIDRα2-6 domains of PfEMP1 bind to CD36 (38), and CIDRα1 binds to EPCR on endothelial cells (39).

## Host Virulence Factors

On the host side of malaria pathology, activated endothelial cells play a significant role in mediating cerebral manifestations. Endothelial cells are activated permanently upon *Pf* infection and sequestration of iRBCs to the vasculature. This chronic activation leads to dysregulation in the endothelial barrier function and brain oedema (40).

### Activation of the Brain Endothelium

Brain endothelial cells are activated during the first phase of *Pf* infection, even before the sequestration of iRBCs. The exact mechanism of early endothelial cell activation is unknown, but it is hypothesized that soluble factors from iRBCs such as *Pf* histidine-rich-protein 2 (PfHRP2) might activate brain endothelial cells (41). PfHRP2 has been suggested as a prognostic marker for CM as plasma levels increase with malaria severity (42). However, studies outside the African continent could not confirm the correlation between plasma levels and disease complications (43).

Changes in the bioavailability of nitric oxide (NO), being the principal protector of endothelium homeostasis, have been reported in children with *Pf* malaria (44). However, inhaled NO as adjunctive therapy for severe malaria proved insufficient for change of outcome (45). Another vasoactive substance elevated in the plasma of malaria patients is endothelin-1 (ET-1) (46). This peptide is among the most effective vasoconstrictive peptides in the human body, but whether ET-1 is involved in vasculopathy in cerebral manifestations of malaria remains unanswered.

Under hypoxic conditions, after vasoconstriction due to sequestration of iRBCs, vascular endothelial growth factor-A (VEGF-A) is released by endothelial cells (47). VEGF-A binds to its receptor (VEGFR2) on endothelial cells, inducing vascular permeability. On the other hand, VEGF-A exerts protective, anti-apoptotic effects in endothelial and neuronal cells. As reviewed elsewhere, the role of VEGF-A remains controversial (48). However, a study investigating serum samples from malaria patients showed significantly lower levels of VEGF-A in cerebral malaria non-survivors, pointing instead to a protective effect of VEGF-A in cerebral complications of malaria (49). Similar protective effects on the vascular brain endothelium are mediated by angiopoietin-1 (Ang-1) and its receptor Tie-2. Ang-1 is essential for endothelial quiescence but can be blocked by Ang-2, which renders a natural competing antagonist for Ang-1 regarding the binding to their shared Tie-2 receptor. Ang-2 destabilizes existing vessels as part of the initiation of angiogenesis, the formation of new blood vessels (50), and is released from activated endothelial cells. Elevated levels of Ang-2 are found in severe and cerebral malaria (47, 51). Although anti-malarial therapy decreases Ang-2 levels (52), direct targeting of the Ang-2/Tie-2 pathway remains challenging and has been reviewed elsewhere (53).

Many of these peptides from endothelial activation have been suggested as possible biomarkers to predict the fatal outcome of CM. However, currently, there is no test available to confirm the diagnosis of cerebral manifestations prior to the appearance of clinical symptoms.

### Blood-Brain Barrier Disruption

The blood-brain barrier (BBB) comprises brain endothelial cells connected by tight junctions formed by transmembrane proteins occludin, claudin and zonula occludens protein-1 (ZO-1). Endothelial cells are in contact with surrounding pericytes and astrocytes situated in the perivascular space. Together, these cells provide a highly functional barrier between the blood and the brain interstitial fluid. In cerebral malaria, an impaired barrier function allows leakage of plasma proteins and fluids into the perivascular space causing vasogenic oedema and brain swelling (6). One reason for the disintegration of the BBB is the decrease in endothelial tight junction proteins occludin and ZO-1 (54). Another reason for the destruction of the BBB lies in the apoptosis of endothelial cells caused by a hyperinflammatory environment upon lymphocyte sequestration.

### Cytokine Mediated Inflammation of the Brain

Numerous studies showed that serum levels of the pro-inflammatory cytokine TNF-α were higher in CM than in severe forms of malaria in children and adults (55). Nevertheless, a more recent study showed that brain swelling of children suffering from CM is independent of peripheral plasma cytokine levels (56), and therapy with a monoclonal antibody against TNF-α did not improve survival in CM patients (57).

Likewise, IFN-γ is released from immune cells like CD4+ and CD8+ T cells, natural killer cells and γδ-T cells during malaria infection. IFN-γ is a potent activator of macrophages, increasing their phagocytosis activity, vital in the early control of parasite growth. On the other side, IFN-γ induces brain endothelium activation and an increase of adhesion molecules (58). However, targeting the IFN-γ pathway as adjunctive therapy for CM is questionable as this important cytokine is involved in many distinctive processes important for gaining immunity against malaria (59).

### Chemokine Induced Migration to the Brain

Besides promoting the expression of adhesion molecules in the brain vasculature, IFN-γ is active in up-regulating CXCL10 released from endothelial cells (60). Confirming, CXCL10 has been described as a biomarker of CM and a predictor of mortality (61). The receptor to which CXCL10 binds is CXCR3, which is predominately expressed on immune cells such as CD4+ and CD8+ T-cells (62). Recently, the adhesion of CD8+ T cells to the brain vascular endothelium was shown to be involved in manifestations of human CM for the first time (63).

### CD8+ T Cells Sequestration

From murine cerebral malaria research, we know that T cells play a crucial role in the experimental cerebral malaria (ECM) model, and functional studies using neutralizing antibodies or T cell-deficient mice have demonstrated a significant role of CD8+ T cells in inducing brain damage (64). However, evidence for the involvement of CD8+ T cells in human CM has long been missing. Recent studies have investigated the presence of CD8+ T cells in human CM post-mortem sections. A first study successfully showed the presence of CD8+ T cells in the brains of children who died from malaria. However, a clear correlation to cerebral manifestations could not be drawn (65). Finally, Riggle et al. provided evidence for the significant involvement of CD8+ T cells in the human setting by investigating 31 brains of children who had died from CM, using multiplexed histology (63). Interestingly, the presence of CD8+ T cells was correlated with the number of iRBCs within the lumen of brain veins. Additionally, sequestered CD8+ T cells showed positive staining for the cytolytic protease granzyme B (GrB) (63). As enzymes such as GrB (66) and perforin (67) are responsible for apoptosis of endothelial cells in ECM, the findings presented by Riggle et al. indicate a similar mechanism in human CM and underline the relevance of the murine malaria model.

## Immunological Aspects of CM Pathology

**Figure 1:**
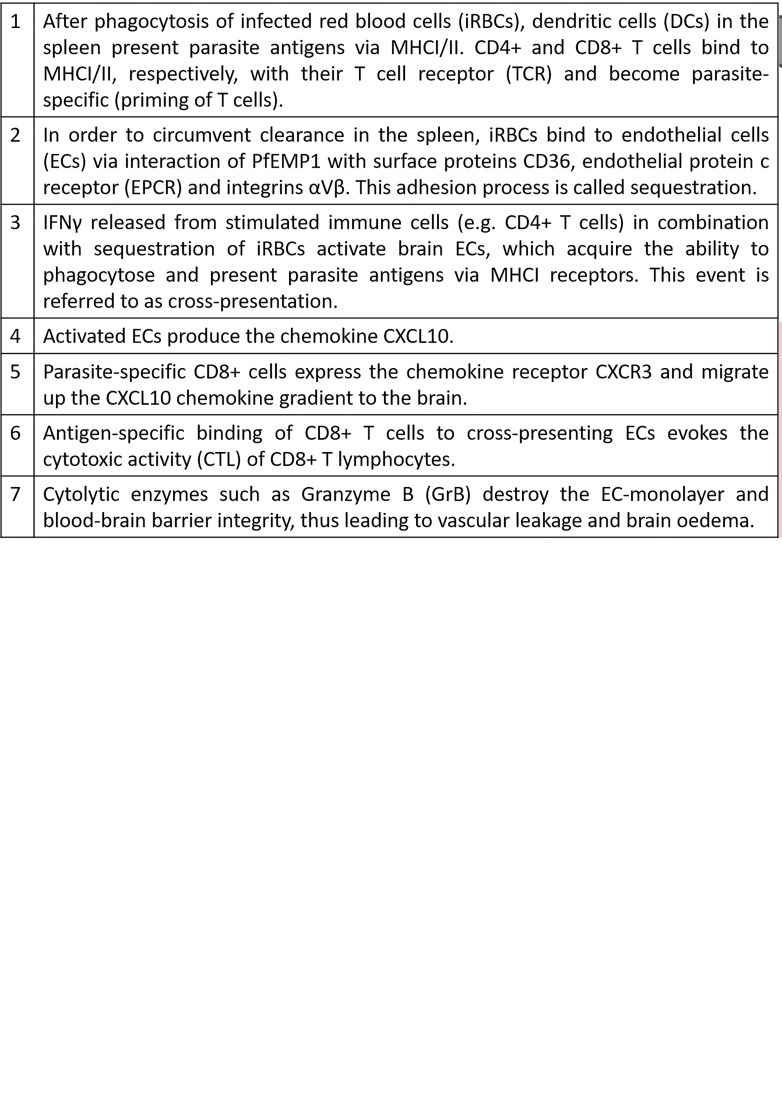
CD8+ T cell dependent CM pathology.

CD8+ T cells recognize pathogens through major histocompatibility complex I molecules (MHCI) on the surface of antigen-presenting cells or infected cells and contribute, therefore, to clearance and immunity against intracellular pathogens. However, as erythrocytes lack MHCI receptors, CD8+ T cells do not recognize *Pf* infected RBCs and are therefore unable to add to clearance of blood-stage infections. Instead, CD8+ T cells are suspected to be the main drivers for cerebral pathology in humans and experimental mouse models (68, 69). One important step for enabling cytotoxic CD8+ T cells (CTLs) to interfere with brain ECs, is a specific process called cross-presentation. As a result of chronic activation of ECs by sequestration of iRBCs in combination with high IFNγ levels, ECs start to phagocytose parasites (e.g. free merozoites) and present these antigens *via* their major histocompatibility complex class I (MHCI) (70, 71). In **Figure 1**, we compile the current knowledge of all the stepwise mechanisms which, as we propose, may lead to CM in non-immune humans.

## Experimental Cerebral Malaria

The mouse model to examine CM named experimental cerebral malaria (ECM) is studied with C57BL/6 mice infected with *Plasmodium berghei ANKA (PbA)*. In this mouse model, RBCs bearing asexual forms of the parasite are injected intraperitoneal (10^6^ infected RBCs) or intravenously (10^4^ infected RBCs). As asexual forms do not enter hepatocytes, the liver stage of the disease is circumvented, and the blood stage is visible in blood smears after approximately three days. Mice show signs of neurological symptoms such as ataxia, convulsions or paralysis starting from day four and die between days 6-8. Mice that do not develop cerebral manifestations die from hyper-parasitemia and anaemia at later time points (day 14 to 21) (72). Similarly to human CM, brain pathology includes dysfunction of the BBB, brain haemorrhaging and brain swelling (68). In addition, mice with neurological symptoms treated with anti-malarial drugs survive with long-lasting cognitive deficits (73). The most significant difference between the murine model and human CM is the extent of iRBCs sequestration in the brain’s microvasculature (74) due to the lack of molecules on RBCs infected with PbA. Therefore, the malaria mouse model is mainly used to study host virulence factors of malaria. However, the presence of parasites in the brain vasculature is also critical for ECM (75, 76). Several studies searched for functional equivalents of PfEMP1 in mouse and non-*falciparum* human *Plasmodium* species (77, 78). Two proteins necessary for the transport of PfEMP1 to the erythrocyte surface, *SBP1* and *MAHRP1*, are evolutionarily conserved between *Plasmodium* species, and their orthologues have been identified in *Plasmodium berghei*, although the transported surface protein is not known (79–81).

## Mouse Models of CM and Their Translational Potential

In experimental malaria models, multiple pathways have been classified as essential for developing CM. After the deletion of specific genes, several knockout mice infected with *PbA* showed resistance against cerebral manifestations of malaria. The identified genes can serve as potential therapeutic targets. Many of the involved genes discussed hereafter encode proteins that function in leukocyte migration to the brain and modulate T-cell effector functions. However, the limitations of the experimental murine model for translational research will also be discussed in the following passage.

### Interferon-Gamma

Cytokines such as interferon-gamma (IFNγ) and its receptors (IFNγR) seem crucial for CM development. In mouse models, IFNγR2 knockout proved protective against CM, whereas specific knockout on T cells (CD4-Cre^+^ IFN-γR2^flox/flox^) did not prevent cerebral symptoms and early death (82). A probable explanation for the survival benefits in an IFNγ deficient system is that this cytokine is involved in activating the brain endothelium, enabling the cross-presentation of parasite proteins by endothelial cells. Therefore, endothelial cells act as antigen-presenting cells (APCs) and are thus recognized by cytotoxic T cells and destroyed. As a result, the endothelial layer gets leaky and infiltrating fluid causes brain swelling (proposed in **Figure 1**).

Outside the model of ECM, however, IFNγ is essential for a functional immune system, and its stimulation of innate and adaptive immune responses is crucial to attack hepatic stages of *Pf* during the early stage of infection (83). Therefore, blocking IFNγ during a *Pf* infection might do more harm in the long run than prevent cerebral complications.

### Tumour Necrosis Factor-Alpha

Tumour necrosis factor receptor 2 (TNFR2) deficient mice were resistant to CM pathology and maintained BBB integrity during infection with *PbA* (84). Accordingly, in the human setting, TNFα has been suggested to mediate the sequestration of iRBCs by upregulation of adhesion molecules on endothelial cells and induce the fatal inflammatory cascade together with IFNγ and nitric oxide (85).

Instead, Lymphotoxin alpha (LTα), which binds the same receptors as TNFα (TNFR1 and TNFR2), might be a more specific target and control late-stage inflammation to prevent neurological complications. LTα knockout animals and LIGHT-lymphotoxin beta receptor (LTβR) deficient mice were protected from CM (86), and LTβR-/- mice showed reduced lymphocyte recruitment to the brain resulting in a survival benefit (87).

However, the translational potential of TNFα and LTα for human CM is questionable, as, similar to IFNγ, TNFα and its receptors are associated with gaining immunity against malaria (88).

### Interleukins

Interleukins (IL) are other cytokine family members playing a role in cerebral malaria complications. For instance, prophylactic treatment with IL-4 increased survival time in *PbA* infected mice by reducing parasitemia by stimulation of Th2 CD4+ T cells and the phagocytic system. However, IL-4 treatment was only effective as a preventive strategy, whereas starting therapy on day five after infection did not abrogate CM fatality (89). However, knockout of the interleukin-4 receptor alpha (IL-4Rα) specifically on dendritic cells (DCs) resulted in decreased numbers of cytotoxic CD8+ T cells in the brain (90). In human studies, increased serum levels of IL-4 are associated with severe and cerebral forms of malaria (91), probably by increasing the fatal inflammatory response.

Interleukin-12 receptor beta2 (IL-12Rβ2) deficient mice are protected against cerebral complications, whereas IL-12-p40 deficient mice show similar susceptibility as wild-type animals (92), suggesting that ECM induction through IL-12Rβ2 can occur independently of IL-12 ligands. Interestingly, IL-12Rβ2 primarily occurs on activated T cells and NK cells, emphasizing the role of these cells in ECM pathology. In human CM, decreased plasma levels of IL-12 occur in severe childhood malaria (93). Unfortunately, no study differentiated between severe and cerebral pathologies of malaria to give data on IL-12 involvement in human brain pathology.

The Interleukin-33 receptor (ST2) is expressed on brain endothelial cells, and ST2 knockout mice are protected from CM due to less cytotoxic CD8+ T in the brain microvasculature (94).

### Adhesion Molecules

The role of the adhesion molecule ICAM1 in ECM development has long been an undoubted truth as ICAM^-/-^ mice are protected from brain pathologies (95). With newer technologies enabling ICAM1 knock-down selectively on endothelial cells (ECs), this theory has become questionable, as these EC-specific knockout mice still developed CM with cellular sequestration independent from ICAM-1 expression on cerebral microvasculature (96). Recently, PfEMP1 A-Type ICAM-1-binding domains have been shown to be not associated with CM in children (97). Instead, the role of PfEMP1 binding to endothelial protein C receptor (EPCR) was significantly linked with brain swelling (98).

Integrin αDβ2 on the surface of lymphocytes is involved in adhesion to brain vessels (99), a critical step in ECM development. In a preclinical study investigating PfEMP1 interacting partners, integrins αVβ3 and αVβ6 have been shown to bind to the DBLδ_D4 domain of a specific parasite line expressing the single var gene PFL2665c (100). Therefore, the authors have suggested that endothelial cells expressing αVβ3 and αVβ6 integrins potentially add to the sequestration of iRBCs *via* the DBLδ_D4 domain of PfEMP1.

CD36 (cluster of differentiation 36) is a membrane protein found on the surface of ECs and has been described as essential for cytoadherence of iRBCs in ECM, although survival of CD36 knockout mice was not improved (101). In human CM, polymorphisms of CD36 are associated with protection from neurological complications (102), and CD36 is a common target of the PfEMP1 protein on iRBCs for adherence to endothelial receptors (103).

### Chemokines

Chemokine receptor CXCR3^-/-^ mice are protected from fatal ECM by decreased infiltration of perforin-positive CD8+ T cells to the brain. The results suggest that CXCR3 is necessary for the migration of CD8+ cells to the brain (104). The ligands of the CXCR3 receptor, IP-10 (CXCL10) and Mig (CXCL9), however, did only partially protect mice from fatal CM when genetically knocked out (105). In patients, CXCL10 serum levels have been significantly associated with a high risk for cerebral complications (61) and suggested as prognostic biomarkers for severe and cerebral complications of malaria (106).

### Transcription Factors

When the transcription factor Batf3 is knocked out in mice, animals lack a specific DC subset necessary for T cell priming. As a result, Batf3^-/-^ mice show decreased cytolytic active CD8+ T cells and are protected from developing CM (107). Batf3 is a potential target for immunotherapy in humans, although its impact on CM has not been investigated so far (108).

### Lipoproteins

Apolipoprotein E (ApoE) is the dominant apolipoprotein in the brain, and ApoE^−/−^ mice are protected against the development of ECM through decreased sequestration of parasites and T cells within the brain. Additionally, treating mice with the ApoE antagonist heparin octasaccharide significantly decreased ECM incidence (109). Whether heparin-based therapies might be promising for reestablishing blood perfusion in congested brain microvasculature, however, is unclear, as bleedings are a possible complication in the neurological complex of CM.

### Protein-Kinases

Protein kinase C-theta (PKC-theta) deficient mice do not show neurologic symptoms typical for CM, such as abrogated cerebral microcirculation or brain ischemia. Interestingly, recruitment and activation of CD8+ T cells were reduced in the brain of resistant mice (110). Further investigation with specific pharmacological inhibitors of the PKC-theta pathway may present a new treatment strategy that needs to be investigated.

## Conclusion

Summing up the latest research data from mouse and human CM studies, the importance of lymphocyte sequestration in the brain vasculature has achieved objective evidence. Although substantial progress has been made in elucidating the cause of death in CM, specific treatment is still missing, and solutions are not within sight. For this reason, research regarding targets, which are drugable in the human setting, is urgently needed, and focus should be concentrated on the development of adjunctive therapies for treating and preventing the potentially fatal evolution into CM.

## Author Contributions

KA-S wrote the manuscript, performed literature research and prepared the table and figure. PL reviewed the manuscript and added an essential part due to his expertise in malaria research and neurology. ES reviewed the manuscript and added essential parts due to his expertise in malaria research and neurology. GB revised the manuscript and added an essential part due to his expertise in T cell biology. All authors contributed to the article and approved the submitted version.

## Funding

This study was supported by the Austrian Science Fund TAI80-B to KA-S.

## Conflict of Interest

The authors declare that the research was conducted in the absence of any commercial or financial relationships that could be construed as a potential conflict of interest.

## Publisher’s Note

All claims expressed in this article are solely those of the authors and do not necessarily represent those of their affiliated organizations, or those of the publisher, the editors and the reviewers. Any product that may be evaluated in this article, or claim that may be made by its manufacturer, is not guaranteed or endorsed by the publisher.
